# Suspected neurological toxicity after oral application of fluralaner (Bravecto®) in a Kooikerhondje dog

**DOI:** 10.1186/s12917-019-2016-4

**Published:** 2019-08-07

**Authors:** Daniela Gaens, Christoph Rummel, Martin Schmidt, Melanie Hamann, Joachim Geyer

**Affiliations:** 1 Institute of Pharmacology and Toxicology, Biomedical Research Center Seltersberg, Justus Liebig University Giessen, Schubertstr. 81, 35392 Giessen, Germany; 2 Institute of Veterinary Physiology and Biochemistry, Justus Liebig University Giessen, Giessen, Germany; 3 Department of Veterinary Clinical Sciences, Clinic for Small Animal-Surgery, Faculty of Veterinary Medicine, Justus-Liebig-University Giessen, Giessen, Germany

**Keywords:** Bravecto, Fluralaner, Dog, Adverse drug reaction, Neurological toxicity, MDR1 mutation

## Abstract

**Background:**

Although the new isoxazoline drug fluralaner (Bravecto®) is generally well tolerated in dogs, adverse drug reactions involving neurological dysfunction occurred in individual dogs. However, most of these cases are documented inadequately and none of them is reported and discussed in the literature. As isoxazoline drugs target neuronal chloride channels with a clear preference for invertebrates, they are considered to have a good safety profile. However, pharmacodynamic effects in the nervous system of vertebrates cannot be ruled out completely.

**Case presentation:**

A seven-month-old female Kooikerhondje dog was treated with Bravecto® at the recommended dose. About 24 h after administration, the dog exhibited signs of neurological toxicity, including generalized ataxia, myoclonic jerks, tremor of head and body, muscle twitching and oral dysphagia. All symptoms were transient and the dog fully recovered without any treatment after 10 h.

**Conclusion:**

This case report describes transient occurrence of neurological dysfunction after administration of Bravecto®. It may help to better classify adverse drug reactions after application of isoxazoline drugs and documents a good prognosis even after occurrence of severe neurological dysfunction in the present case.

**Electronic supplementary material:**

The online version of this article (10.1186/s12917-019-2016-4) contains supplementary material, which is available to authorized users.

## Background

Fluralaner (syn. A1443) is a novel systemic ectoparasiticide and belongs to the class of isoxazolines, also including afoxolaner, lotilaner and sarolaner [1, 2]. Isoxazolines are potent inhibitors of gamma-aminobutyric acid (GABA)- and glutamate-gated chloride channels in the nervous system of invertebrates [3]. Thereby, they result in uncontrolled neural activity and finally death of insects and acarines [4]. Binding to and blocking of phylogenetically related vertebrate pentameric ligand-gated chloride channels (i.e. GABA and glycine receptors) is expected to be low [5]. Nonetheless, signs of neurological toxicity, including tremor, ataxia and convulsions have been observed in a target animal safety study for sarolaner at overdose [6]. Although this was only reported for sarolaner so far, in general all isoxazoline drugs may have the potential for neurological toxicity in vertebrates as they have the same mode of action.

One of the approved pharmaceuticals containing fluralaner is Bravecto®, which is available as chewable tablet for the treatment of tick and flea infestations in dogs [7]. Although Bravecto® chewable tablets have been well tolerated in clinical studies in dogs within the framework of drug approval, more recently several cases of individual adverse drug reactions after application of Bravecto® were reported. Some of them included severe neurologic adverse events such as tremors, ataxia and seizures [8]. The current CVMP (Committee for Veterinary Medicinal Products of the European Medicines Agency) statement notes that convulsions and lethargy have been reported very rarely in spontaneous pharmacovigilance reports and emphasizes that Bravecto® should be used with caution in dogs with pre-existing epilepsy. Nevertheless, the CVMP still considers the use of Bravecto® chewable tablets to be acceptably safe [9]. The case report presented here describes and characterizes transient occurrence of neurological dysfunction in a Kooikerhondje dog and has a possible causal relationship with the administration of Bravecto® chewable tablets.

## Case presentation

The case report is on a female Kooikerhondje dog with parental animals approved by the German Kooikerhondje Club (DCK) for breeding and tested free of Hereditary Necrotising Myelopathy (ENM) and von Willebrand’s disease according to the club’s regulations. The dog receives the recommended amount of pellet food (Platinum©) and was 7 months old on the day of the Bravecto® treatment. The vaccination status included rabies, distemper, parvovirosis, infectious canine hepatitis, leptospirosis and canine parainfluenza virus. Deworming was regularly performed at the recommended dosage with the last treatment by Milpro® (containing milbemycin oxime and praziquantel) one month before the Bravecto® administration. At the age of 13.5 weeks, the dog already received a first application with Bravecto® and no adverse drug reactions were observed for this previous treatment. At 5 and 6 months of age, the dog suffered from mild gastrointestinal symptoms with vomiting and mild diarrhea, which resolved after treatment with amoxicillin, metoclopramide and a gastrointestinal diet (Royal Canin™). Clinical and X-ray examination did not show any sign of obstipation or ileus but two small foreign bodies that were subsequently discarded the next day by defecation. Blood analyses did not reveal any abnormal parameters but slightly elevated inorganic phosphate plasma levels. The dog did not show any signs of neurological disorders at any time before or after the clinical case described here. In general, the dog’s state of health was good and without any abnormalities at the time of drug administration.

Bravecto® tablets were provided at the recommended dosage of 250 mg fluralaner (lot: U196A02). The dog had about 9 kg of body weight at the time of treatment (dosage ~ 28 mg/kg). The dog had received 40 g of pellet food (Platinum©) approximately 1.5 h prior to treatment. First signs of neurological toxicity were observed at approximately 24 h after treatment. The dog showed intermittent inability to walk and signs of disturbed equilibrium. The clinical signs worsened within an hour. Controlled movements or walking were hardly possible. The dog was not able to stand up on its own (see Additional file 1). He showed a mild wide-based stance, generalized symmetric ataxia in all four limbs, with mixed hypometric and hypermetric limb movements, and intermittent head tilt to the left. Nystagmus was not observed. Proprioception, cranial nerve examination and spinal reflexes were normal. Clinical signs suggested impaired vestibular-cerebellar functions. During the night, the dog slept in his kennel with shallow breathing and hardly reacted after vocal or tactile stimulation. The next morning, the dog showed oral dysphagia in the manner of dropping food from the mouth, but was able to ingest the food after several attempts. Gait problems improved progressively and completely disappeared around 10–11 h after their onset.


**Additional file 1:** **Supplementary video material.** Kooikerhondje dog showing signs of neurological toxicity after application of Bravecto® (fluralaner). (MP4 1890 kb)


## Discussion

Fluralaner is a potent acaricide and insecticide from the isoxazoline drug class. A single dose of Bravecto® administered orally to dogs provides at least twelve weeks of flea- and tick-control [10]. This prolonged activity of the active compound may be explained by its pharmacokinetic properties. It is readily absorbed after single-dose oral administration reaching C_max_ within one day, shows long half-life, long mean residence time, relatively high apparent volume of distribution, and low clearance with enterohepatic re-circulation [11].

Fluralaner inhibits arthropod glutamate-gated chloride channels (GluCls) and GABA-gated chloride channels (GABACls) [12], which structurally belong to the class of so-called Cys-loop receptors [13, 14]. Arthropod Cys-loop receptors are targeted by many different antiparasitic drugs, including fipronil, ivermectin, and fluralaner [2]. Single experiments on housefly-head and rat-brain membranes as well as on recombinantly expressed individual receptors point to a significantly lower binding affinity of fluralaner to vertebrate receptors compared with arthropod receptors [1, 5, 15]. Nevertheless, it cannot be excluded that fluralaner also interacts with one of the vertebrate Cys-loop receptors in vivo, which are highly or even exclusively expressed in the central nervous system [16, 17]. Based on the molecular pharmacology of fluralaner, it has to be discussed if the neurological dysfunction seen in the Kooikerhondje dog in the present case may result from blocking of one of these receptors, which would explain the occurrence of generalized ataxia, myoclonic jerks, tremor of head and body, muscle twitching and oral dysphagia. Of note, the onset of the neurological dysfunction was reported exactly at the time of the expected C_max_ of fluralaner in dogs [11].

However, as fluralaner has a generally good safety profile and neurologic adverse events only occur in very rare cases, individual factors increasing the drug concentration in the brain or increasing the susceptibility of CNS Cys-loop receptors may come into account. However, currently only few factors are known that affect the pharmacokinetic profile and brain penetration of fluralaner. One of them is a defect of the multidrug resistance (MDR1) drug efflux carrier at the blood-brain barrier (commonly referred to as *MDR1* nt230(del4)), frequently present in Collie, Australian Shepherd, Shetland Sheepdog, Longhaired Whippet, White Swiss Shepherd and some other breeds [18], which leads to increased drug penetration into the brain [19]. This can provoke neurological toxicity in *MDR1* mutant dogs even at standard dosage, as it is well known for drugs like ivermectin [20]. Very recently, significantly increased brain penetration of fluralaner was demonstrated in an *mdr1* mutant mouse model, indicating that MDR1-mediated drug efflux normally prevents fluralaner entry into the brain [21]. Although this breed is not suspected to carry this gene mutation, MDR1 genotyping of the Kooikerhondje dog was performed and revealed an *MDR1* intact *MDR1*^*+/+*^ genotype, excluding increased brain penetration of fluralaner due to *MDR1* nt230(del4) mutation in the present case. Nevertheless, occurrence of other mutations in the *MDR1* gene or related drug efflux carrier at the blood-brain barrier can not be excluded. Furthermore, the premedication of the dog with milbemycin oxime plus praziquantel one month prior to Bravecto® treatment may have increased brain penetration of fluralaner, e.g. by inhibition of MDR1-mediated fluralaner efflux by the premedication via drug-drug interaction. However, fluralaner was shown to be safe when administered concurrently with milbemycin oxime + praziquantel [22].

Furthermore, a combination of different individual factors might have affected the bioavailability, pharmacokinetics and brain penetration of fluralaner in the Kooikerhondje dog and thus may have provoked the neurological dysfunction. These could include inter-individual variability in gastrointestinal pH, time of gastric emptying, duration of intestinal transit, plasma protein binding as well as age and sex of the animal [23, 24]. Additionally, breed-related differences in body constitution might play a role for the margin of safety of drugs [25]. However, in pivotal effectiveness studies of fluralaner [7], various dog breeds and mongrels were included and no obvious differences in drug safety across breeds were noted.

Another factor influencing the pharmacokinetics of fluralaner might be the prandial state of the dog at the time of drug application, as it is well known that feeding affects gastrointestinal physiology and thereby may affect drug absorption and bioavailability [26, 27]. A study investigating the influence of concurrent feeding on fluralaner pharmacokinetics revealed that there were no dramatic differences between fasted and fed dogs, but that food significantly increased the bioavailability of fluralaner from the Bravecto® chewable tablets [28]. As the recommendation of the manufacturer is to administer Bravecto® at or around feeding, as it was performed in the Kooikerhondje dog, feeding is suggested to play a minor role in the present case.

Of note, neurological signs were transient and resolved without any treatment in the Kooikerhondje dog. This might be explained by increased drug levels in the brain around the fluralaner plasma T_max_, reached within one day on average in beagle dogs after administration of Bravecto® [11]. In this case, improvement of the dog’s condition and disappearance of neurological dysfunction might simply be explained by drug elimination and decline of the relevant drug concentration in the brain. Therefore, in the current case no particular treatment was necessary and cannot be recommended until the molecular mechanisms behind the observed signs of neurological toxicity in dogs are better understood. Based on the supposed mechanism of blocking Cys-loop receptors, GABA_A_ receptor agonists such as benzodiazepine drugs or propofol might be a treatment option [29], but until now there is no clinical experience with that at all. Furthermore, administration of an intravenous lipid emulsion (ILE) might be helpful. This treatment was previously administered in cases of intoxications with lipophilic drugs, such as ivermectin in dogs [30]. In order to prevent enterohepatic re-circulation of fluralaner, administration of activated charcoal might also be useful. Overall, depending on the severity of the neurologic adverse events and the patient’s general health condition, symptomatic treatment and supportive care are recommended as it was performed in the present case.

## Conclusions

Although Bravecto® is generally well tolerated in dogs, based on the molecular pharmacology of fluralaner, pharmacodynamic effects in the nervous system of vertebrates cannot be ruled out completely. In the present case, a Kooikerhondje dog was treated with Bravecto® at the recommended dose and the onset of signs of neurological toxicity was around the C_max_ of fluralaner. This indicates a possible causal relationship between drug treatment and the neurological dysfunction. All symptoms were transient and the dog fully recovered without any treatment after 10 h. This case report may help to better classify adverse drug reactions after application of isoxazoline drugs and documents a good prognosis even after occurrence of severe neurological dysfunction in the present case.

However, several points remain unclear. As Bravecto® treatment was tolerated by the Kooikerhondje dog in a previous treatment, general drug hypersensitivity can be excluded. Additional factors which limit drug tolerability are either unknown so far or are not relevant for the present case (feeding status, MDR1 genotype). Furthermore, it is unknown if milbemycin oxime plus praziquantel treatment one month before the Bravecto® administration can provoke relevant drug-drug interactions. Finally, occurrence of neurological dysfunction due to other reasons, independent from Bravecto® treatment, cannot be ruled out completely.

In the future, research is needed to elucidate potential molecular targets of fluralaner in the brain of vertebrates and to identify any factor that limits drug tolerance. Furthermore, treatment options should be evaluated to support individual dogs with appearance of neurologic adverse events similar to the present case. In general, the dog owner should be informed about possible adverse drug reactions after treatment with Bravecto® and an individual benefit-risk assessment must be performed. Particularly, fluralaner should be used with caution in dogs with pre-existing epilepsy.

## Data Availability

This case report contains all relevant data. An additional video file is submitted together with the manuscript.

## Abbreviations

CNS: Central nervous system
CVMP: Committee for Veterinary Medicinal Products
GABA: Gamma-aminobutyric acid
GABACls: GABA-gated chloride channels
GluCls: Glutamate-gated chloride channels
MDR1: Multidrug resistance gene 1
